# Prader-Willi Syndrome: Obesity due to Genomic Imprinting

**DOI:** 10.2174/138920211795677877

**Published:** 2011-05

**Authors:** Merlin G Butler

**Affiliations:** Departments of Psychiatry & Behavioral Sciences and Pediatrics, Kansas University Medical Center, Kansas City, Kansas, USA

**Keywords:** Prader-Willi syndrome, Angelman syndrome, genomic imprinting, deletion, maternal disomy, clinical presentation and differences, genetic subtypes.

## Abstract

Prader-Willi syndrome (PWS) is a complex neurodevelopmental disorder due to errors in genomic imprinting with loss of imprinted genes that are paternally expressed from the chromosome 15q11-q13 region. Approximately 70% of individuals with PWS have a de novo deletion of the paternally derived 15q11-q13 region in which there are two subtypes (i.e., larger Type I or smaller Type II), maternal disomy 15 (both 15s from the mother) in about 25% of cases, and the remaining subjects have either defects in the imprinting center controlling the activity of imprinted genes or due to other chromosome 15 rearrangements. PWS is characterized by a particular facial appearance, infantile hypotonia, a poor suck and feeding difficulties, hypogonadism and hypogenitalism in both sexes, short stature and small hands and feet due to growth hormone deficiency, mild learning and behavioral problems (e.g., skin picking, temper tantrums) and hyperphagia leading to early childhood obesity. Obesity is a significant health problem, if uncontrolled. PWS is considered the most common known genetic cause of morbid obesity in children. The chromosome 15q11-q13 region contains approximately 100 genes and transcripts in which about 10 are imprinted and paternally expressed. This region can be divided into four groups: 1) a proximal non-imprinted region; 2) a PWS paternal-only expressed region containing protein-coding and non-coding genes; 3) an Angelman syndrome region containing maternally expressed genes and 4) a distal non-imprinted region. This review summarizes the current understanding of the genetic causes, the natural history and clinical presentation of individuals with PWS.

## INTRODUCTION AND HISTORICAL REVIEW

Prader-Willi syndrome (PWS) is a complex neurodevelopmental genetic condition due to paternal loss of imprinted genes on chromosome 15 and characterized by a range of mental and physical findings including obesity that can be life-threatening [1, 2]. It affects an estimated 350,000–400,000 people worldwide. The Prader-Willi Syndrome Association (USA) estimates that 17,000–22,000 individuals live in the United States [3]. Therefore, PWS is relatively common with an estimated prevalence worldwide in the range of 1 in 10,000 to 30,000 individuals [1, 4]. PWS is considered one of the more common disorders seen for genetic services and presents in all races and ethnic groups [2, 5, 6]. Most cases are sporadic; however, in some families a defective control of differentially expressed genes from the chromosome 15q11-q13 region is present causing PWS through errors in processing of genomic imprints in the PWS child and carried by the father. This error can be contributed by the father’s mother and lead to recurrence of another child with PWS. The risk may be as high as 50% [7, 8].

PWS arises from lack of expression of paternally inherited genes known to be imprinted and located in the 15q11-q13 region. Genomic imprinting is an epigenetic phenomenon whereby the phenotype is modified depending on the sex of the parent contributing the gene allele [9] and arises from epigenetic changes when expression of genes is controlled without changing the DNA sequence. It is reversible during gametogenesis. The regulation of gene expression is usually through DNA methylation. The control of expression of imprinted genes is dependent on the parent of origin with mono-allelic gene expression of either the maternal or paternal allele for a particular imprinted locus or gene.

Approximately 70% of individuals with PWS are caused by a non-inherited (i.e., *de novo*) deletion in the paternally derived chromosome 15q11-q13 region. The remaining PWS individuals (about 25%) result from maternal disomy 15 (i.e., both chromosome 15s from the mother and no paternal chromosome 15), genomic imprinting defects due to microdeletions or epimutations of the imprinting center located in the 15q11-q13 region in less than 3% of cases, or chromosome 15 translocations or rearrangements [1, 2, 6, 7, 10, 11]. PWS and Angelman syndrome, an entirely different clinical disorder involving the same chromosome region, were the first examples of errors in genomic imprinting in humans [10, 12].

The cardinal features of PWS include infantile hypotonia and feeding difficulties due to a poor suck, hypogonadism and hypogenitalism in both males and females, hyperphagia and onset of obesity in early childhood, short stature due to growth hormone deficiency, small hands and feet, mild intellectual disability (average IQ of 65), behavioral problems including skin picking, temper tantrums, stubbornness and a particular facial appearance. The facial findings include a small upturned nose, narrow bifrontal diameter with almond-shaped eyes, down-turned corners of the mouth with sticky salivary secretions and generally lighter skin, hair and eye color than other family members [1, 2, 6, 13-15]. The features of PWS were first documented in an adolescent female by J. Langdon Down in 1887 [16], but the syndrome went unrecognized until 1956 when Prader, Labhart, and Willi reported nine individuals with similar clinical findings [17].

In 1981, Ledbetter and others [18] used high resolution chromosome analysis to show that more than half of the individuals with PWS they studied had an interstitial deletion of the proximal long arm of chromosome 15 at region q11–q13. Later, Butler and Palmer [19] studied specific polymorphisms found on chromosome 15 and reported that the chromosome 15 deletion was *de novo* in origin and not present in either parent. However, in all cases studied the deletion was donated from the father. This exclusive paternal source of the chromosome deletion was later clarified by molecular genetic techniques. Subsequently, a maternal deletion of the 15q11-q13 region was reported in a separate clinical condition now recognized as Angelman syndrome. Butler and others in 1986 [13] reported clinical differences in those PWS individuals with and without the chromosome 15q11-q13 deletion, particularly hypopigmentation and greater homogeneity in clinical presentation in those individuals with the 15q11-q13 deletion [1, 13, 14].

Using Southern hybridization of polymorphic DNA markers isolated from the 15q11–q13 region, Nicholls, Butler, and others reported in 1989 [10] that individuals with PWS and normal-appearing chromosomes inherited both chromosome 15s from the mother and none from the father. This previously unreported observation was referred to as maternal uniparental disomy 15 or maternal UPD 15. Butler [14] subsequently characterized the hypopigmentation status seen in the majority of individuals with PWS and the 15q deletion. We now know that loss of the P (pigment) gene located in the 15q11-q13 region leads to hypopigmentation. In 2004, Butler and others [20] reported clinical differences between individuals with PWS grouped by two types of deletions; those with the larger typical 15q11-q13 deletion, referred to as Type I, and those with the smaller Type II deletion. The individuals with the Type I deletion had more behavioral and cognitive problems when compared to those with the smaller Type II deletion or maternal disomy 15.

## CLINICAL STAGES AND NATURAL HISTORY

The clinical course of PWS has historically been divided into two distinct clinical stages (early failure-to-thrive and later childhood obesity). With earlier diagnosis and use of growth hormone to treat the growth failure, the clinical course is becoming more variable. The initial phase of the first stage of clinical course development appears unchanged and begins in pregnancy. This stage is noted by decreased fetal activity. After delivery, central hypotonia and a weak cry and suck are present along with a narrow forehead, developmental delay, temperature instability, sticky salivary secretions, and feeding problems often requiring naso-gastric feeding or gastrostomy tube placement. Hypogonadism and underdevelopment of the sex organs in both sexes are noted during this stage [1, 2, 6, 13, 15, 21].

**Fig. (1):**
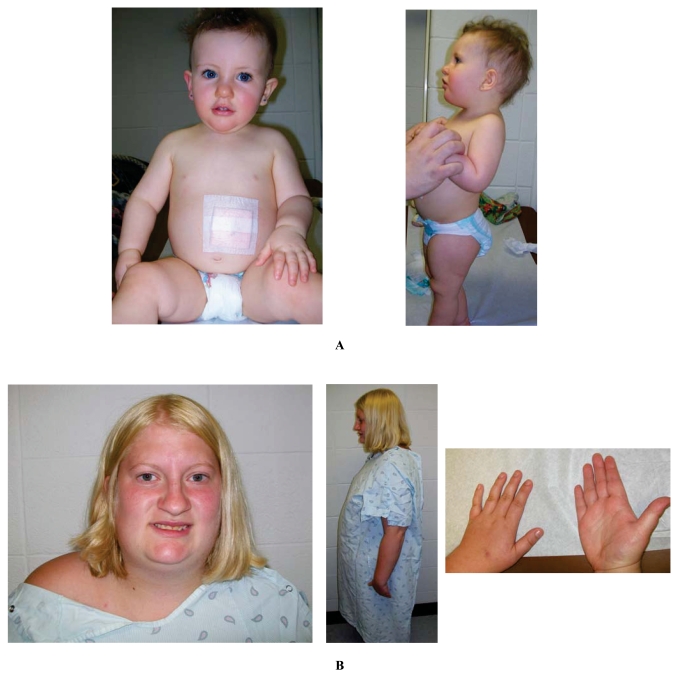
(**A**). Frontal and profile views of a 16 month old female with Prader-Willi syndrome due to maternal disomy 15 and not treated with growth hormone. Note the typical facial features of a narrow forehead, short-upturned nose and downturned corners of the mouth. The gastrostomy site is noted along with central obesity. (**B**). Facial, profile and hand views of an 18 year old female with Prader-Willi syndrome due to the typical 15q11-q13 deletion and not treated with growth hormone. Note the almond-shaped eyes, a narrow forehead, thin upper lip, hypopigmentation, central obesity and small hands.

Typically for the non-growth hormone treated child with PWS, the second stage of clinical course development begins around 2 years of age [13]. This stage is characterized by continued developmental delay and onset of hyperphagia which leads to obesity if not controlled. Speech articulation problems, food foraging, unmotivated sleepiness and physical inactivity are common during this time. Other features include decreased pain sensitivity, skin picking, periods of hypothermia, strabismus, hypopigmentation, scoliosis, sleep apnea, and abnormal oral pathology and dental anomalies [1-3, 6, 21-23]. PWS children are often easy-going and affectionate, but personality problems often develop between 3 and 5 years of age, including temper tantrums, depression, stubbornness and obsessive compulsivity. Sudden outbursts of violence may be observed later along with behavioral changes often initiated by withholding of food. Poor peer interactions, immaturity, and inappropriate social behavior may also occur [6]. These patterns continue into adolescence and adulthood (see Fig. **1**).

### Infancy

Infants with PWS can sit independently at 1 year of age, crawl at 16 months, walk at about 2 years and talk (10 words) at 39 months [6, 13]. Because of generalized hypotonia, neonates with PWS are profoundly floppy. However, brain anomalies are not present based on imaging studies. Hypotonia and decreased muscle mass can lead to respiratory distress and possible asphyxia during illnesses. Central adrenal insufficiency has also been reported in PWS [24].

Most infants with PWS have a weak or absent cry, little spontaneous movement, hyporeflexia, excessive sleepiness, and poor feeding due to diminished swallowing and sucking reflexes which often necessitate gavage feedings and use of special nipples lasting for several months. Thus, a major focus of treatment is addressing the feeding problems and supplying adequate nutrition for growth and development. Growth parameters should be regularly assessed (e.g., weekly) using recently published growth charts for non-growth hormone treated infants with PWS [25] during the first 6 months of life and then monthly until 2 years of age. Calories are adjusted accordingly, but fats should not be restricted even though the non-growth hormone treated infant with PWS requires less than the recommended allowance (often 60% of normal) to avoid rapid weight gain once the failure-to-thrive stage has passed. Vitamin and mineral intake (e.g., calcium) should be monitored by a dietition and supplements given during infancy. Early stimulation programs are also recommended, including occupational and physical therapies. The use of growth hormone is known to alter the clinical presentation [6, 15].

### Childhood

By 18 months of age the feeding pattern in PWS can change radically with an insatiable appetite leading to onset of early childhood obesity. Hyperphagia is a major behavioral problem in PWS and typically lasts over the lifetime. No known pharmacological agent has been effective to treat hyperphagia once it develops.

Global developmental delays and behavior patterns such as temper tantrums, difficulty in changing routines, stubbornness, manipulative behavior, and obsessive-compulsions may become more apparent during childhood. Lying, stealing, and aggressive behavior are common during this time. Although children with PWS can be affectionate, they can also be less agreeable and less open to the introduction of new ideas. Without growth hormone treatment, they are generally less physically active than other children [6].

Endocrine abnormalities such as hypothyroidism, growth and sex hormone deficiency and adrenal problems are reported in PWS and usually recognized early. Enamel hypoplasia and dental caries are frequently seen as well as strabismus and myopia with impaired stereoscopic vision; the latter is more common in maternal disomy 15 [26]. Hypopigmentation also becomes more pronounced. A characteristic body habitus or posture is more noticeable during early childhood with sloping shoulders, central obesity, straight lower leg borders and straight ulnar borders to the hands. Many of these features improve with growth hormone treatment. Small hands and feet and almond-shaped eyes become more apparent during mid-childhood.

Academic achievement is usually impaired during the first 6 years of life. About one-third of children function in the low-normal intellectual range (70–100 IQ) and the remaining in the mild-to-moderate range (50–70 IQ). The average IQ is 65. There are reported differences in behavior, academic achievement, and cognition between those with the chromosome 15q deletion versus those with maternal disomy [27].

Many children begin school in mainstream settings, but the intellectual impairment and potential behavioral problems will present difficulties in progressing through regular classroom settings. Special education and support services are often required. Children with PWS have relatively strong reading, visual, spatial, and long-term memory skills, but have weaker math, sequential processing, and short-term memory skills. Verbal skills may be relative strengths, particularly in those children with maternal disomy, but speech articulation can be a problem requiring speech therapy. A common occurrence in those with the typical deletion is an unusual skill of working with jigsaw puzzles [6, 28].

### Adolescence and Adulthood

Puberty is absent, delayed or incomplete in both males and females with PWS and infertility is present in the vast majority. Menarche may occur but may not be present until 30 years of age. Amenorrhea or oligomenorrhea is usually present and gonadotropin hormone production is low. Adolescents and young adults with PWS usually look younger than their chronological age [29].

Hypogonadism and hypogenitalism occur in the vast majority of individuals with PWS during adolescence. Hypothalamic hypogonadism leads to the abnormal gonadal development and function. Testosterone and estrogen levels are generally low. Cryptorchidism is present in 80-90% of males with a small penis and hypoplastic scrotum. A hypoplastic labia majora and minora and a small clitoris are seen in most females. Penile size increases modestly in many males during the third or fourth decade of life, but testicular size remains small. Treatment of the small penis with topical or parenteral testosterone has been effective, but normalization of genital development is rare. Testes may descend spontaneously in males during childhood and puberty, but often surgical intervention is required. Precocious development of pubic and axillary hair occurs in about 20% of individuals in both sexes as a consequence of premature adrenarche. Beard and body hair are variable, occurring later than normal, but generally sparse. Inadequate voice change occurs in most males. Little information is known about sexual activity in PWS, although individuals with PWS show an interest in affection and establishing relationships. In PWS females, breast development is normal in about one-half of cases with onset between 9 and 13 years of age. Pubic hair is normal in about 40% of females. Although very rare, pregnancies in females with PWS have occurred; there have been no reported pregnancies produced from PWS males [6, 30].

Approximately 90% of individuals with PWS without growth hormone treatment will have short stature by adulthood. The average untreated adult male is 155 cm (61 inches) tall and the adult female averages 147 cm (58 inches) [6]. Growth standards for untreated males and females with PWS have been reported between 2 to 22 years of age [31]. Growth pattern analysis shows that the 50th centile for height in PWS individuals generally falls below the 5th centile for normal individuals by age 12 to 14 years. Height velocity often declines relative to normal due to the lack of a pubertal growth spurt and growth and sex hormone deficiencies. Inverse correlations between Z-scores for linear measurements (e.g., height, hand and foot lengths) and age indicate a deceleration of linear growth with increasing age relative to normal individuals. Short stature is almost always present by the second decade of life without growth hormone replacement [6, 32-35].

Acromicria (small hands and feet) seen during infancy and childhood is more pronounced during adolescence and adulthood. Foot length tends to be more affected than hand length. Scoliosis may also become more pronounced during adolescence and kyphosis may be present by early adulthood. Genu valgus positioning at the knees may also occur [1].

Without intervention, adolescents with PWS may weigh 250 to 300 pounds by their late teens. Beside obesity related complications impacting morbidity and mortality, eating related fatalities do occur, including choking on gorged food and gastric necrosis and rupture [36]. By late adolescence, food stealing and hoarding can be problematic as well as consuming discarded or inedible food items (e.g., frozen food). This often necessitates locking the refrigerator and food cabinets to prevent excessive eating. The eating behavior and complications of obesity can reduce the life expectancy in PWS and cognitive impairments preclude adult independent living arrangements. Behavioral and psychiatric problems often require medical treatment and behavioral management. Psychosis is evident by young adulthood in about 10% of individuals with PWS [37, 38]. Caloric diet restriction is lifelong and important to control obesity. Based on population studies, the death rate in PWS is estimated at 3% per year [39].

The decreased muscle tone and mass in PWS contributes to a lower metabolic rate and physical inactivity with subsequent obesity in adolescents and adults. Improvement is seen with growth hormone therapy. Sleep disorders and respiratory dysfunction in PWS such as hypoventilation and oxygen desaturation are also common from childhood to adulthood and need to be closely monitored before growth hormone treatment. Adolescents have a tendency to fall asleep during the day, particularly when they are inactive, and they do not sleep soundly at night.

Weight and behavior control problems are characteristic of PWS and require involvement of the patient, family members and care providers to address the issues. Interventions to control food consumption include locking refrigerators, limiting the amount of food at home, continual supervision during times of food-related events and providing non-food-related rewards. Strict mealtime regimes are important. Individually tailored exercise programs are also encouraged and should be monitored to reduce weight. Adequate protein during times of caloric restriction should be encouraged to help conserve lean body mass and is particularly needed when growth hormone is prescribed to increase stature and muscle mass. Restricted caloric intake with vitamin and calcium supplementation is generally required by age 2 years and beyond to minimize excessive weight gain and prevent osteoporosis [40].

Historically, weight maintenance in non-growth hormone treated children with PWS requires caloric intake of 8-11 kcal per cm of height per day (by contrast, children without PWS require 11-14 kcal per cm per day for adequate growth) [41]. Weight loss has been documented in children by restricting their caloric intake to 7 kcal per cm per day. Similar guidelines have not yet been developed specifically for adolescents and adults with PWS. Hence, a lifetime commitment and close observation are required by all involved in the care of individuals with PWS, regardless of their age or living arrangement. The obesity associated with PWS results from a chronic imbalance between energy intake and expenditure due to hyperphagia, decreased physical activity, reduced metabolic rate and an inability to vomit. Individuals with PWS have a lower lean body mass compared with controls contributing to reduced energy expenditure. Studies have been conducted to determine the relationship among body composition, activity levels and metabolic rates in PWS. Butler and colleagues [42] demonstrated that individuals with PWS had reduced total energy expenditure and a lower lean body mass compared with obese subjects. In addition, resting energy expenditure was significantly reduced by 16% in the individuals with PWS compared with control subjects.

Theodoro and colleagues [43] studied body composition in PWS individuals between 10 to 49 years. Regional fat and lean mass patterns were characterized and significant differences were found between PWS and obese subjects for lean measures of the arms, legs, and trunk. Total lean mass was significantly lower in PWS than in obese subjects for arms, trunk, and legs, demonstrating an unusual body composition and fatness patterns in PWS characterized by reduced lean tissue and increased adiposity.

Because hyperphagia is the most striking behavior in PWS, Holsen and colleagues [44] used functional magnetic resonance imaging (fMRI) to study the brain’s response to food stimulation images before and after eating in individuals with and without PWS. When viewing food and non-food images before and after eating, the fMRI scans showed that PWS subjects exhibited greater brain activation to food pictures in the post-meal state compared to controls. The activated brain areas included the orbitofrontal cortex, medial prefrontal cortex, insula, hippocampus, and parahippocampal gyrus; several of these regions function to drive eating behavior.

Behavioral and learning problems become more prominent during the teenage years, particularly temper tantrums, self-injury and obsessions. Typical adolescent rebelliousness is often exaggerated, particularly over access to food. Self-injurious behavior is reported in 60% of PWS adolescents and 81% of adults, with skin picking being the most common [45, 46]. Compulsive symptoms have been reported in about 60% of individuals with PWS [47] and occasionally these behaviors worsen during adulthood. In addition, acute psychosis can be seen in about 10% of young adults with PWS, particularly in those with maternal disomy.

Psychotropic agents can be helpful in controlling abnormal behavior, but no specific medication has been universally effective in controlling abnormal behavior or food-seeking behavior. Serotonin agonists have been used to reduce behavioral problems, including temper tantrums and compulsions. However, children and adults with PWS are typically affectionate and outgoing and show a willingness to please others and seek positive attention. When an adult with PWS is surrounded by trained and experienced caregivers, behaviors can be managed.

Adults with PWS have life-oriented goals similar to others entering adulthood, including vocation, independent living and long term relationships. For most persons with PWS, formal education ends between ages 18 and 21. However, a gap often exists between completion of school and entrance into a job-training program. Challenges may be numerous for the adult with PWS and the family members or caregivers. In view of potential health problems such as behavior and hyperphagia, an appropriate environment requires supervised living arrangements with food restrictions. Professional sources knowledgeable about PWS are required for development of training and sheltered employment and housing. Decisions regarding living arrangements and availability of food are usually made unwisely by persons with PWS; therefore it is optimal to have legal guardianship assigned to a parent or other adult.

## PRADER-WILLI SYNDROME: GENETICS

Prader-Willi syndrome is a genomic imprinting disorder due to an epigenetic phenomenon that evolved about 150 million years ago and involves modification of the phenotype of an individual depending on the parent of origin [48]. About 1% of mammalian genes are imprinted and frequently affect growth, development and viability. PWS and other obesity-related and overgrowth disorders such as Albright hereditary osteodystrophy and Beckwith-Weidemann syndrome are examples of errors in genomic imprinting [48].

Epigenetics refers to heritable but reversible regulation of various genetic functions, including gene expression, which is influenced by environmental factors. Nearly all imprinted genes have a CpG-rich differentially methylated region which usually relates to allele repression. Methylation of cytokine bases occurring in the CpG dinucleotides involve key regulatory elements of the genes. Defects in the imprinting center controlling the activity of imprinted genes originate from either parent and can lead to different clinical syndromes. For example, the loss of paternally expressed genes from the 15q11-q13 region leads to PWS with the maternal contribution of this chromosome region normally silenced by epigenetic factors (e.g., DNA methylation). The sister genomic imprinting syndrome, Angelman syndrome, is due to loss of maternal expression of genes in the same region that are normally silenced on the paternal chromosome 15. An ideogram of chromosome 15 and order of genes in the 15q11-q13 region is shown in Fig. (**2**).

**Fig. (2):**
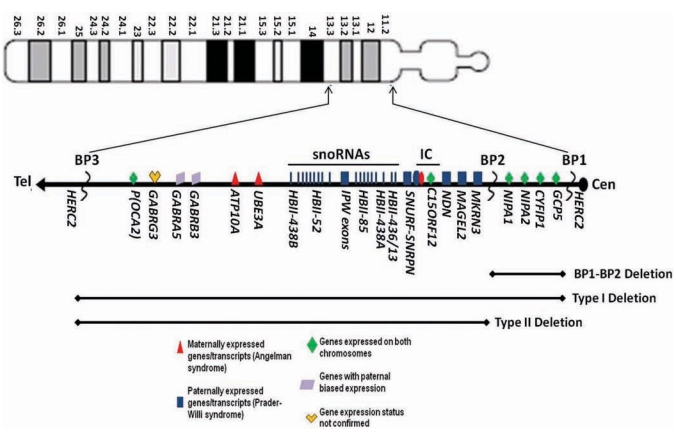
Ideogram of chromosome 15 showing the order of protein-coding and non-coding genes and transcripts in the 15q11-q13 region and location of breakpoints for the typical Type I and Type II deletions. Abbreviations: Cen, centromere; Tel, telomere; BP, breakpoint; IC, imprinting center; snoRNAs, small nucleolar RNAs.

The chromosome 15q11-q13 region contains about 8 million DNA base pairs including a large cluster of imprinted genes and also a non-imprinted domain in which genes are expressed equally on either the maternal or paternal chromosome 15, but with a few genes showing a paternal expression bias [11, 49, 50]. Novel low copy repeat DNA sequences are clustered at or near the two major proximal chromosome breakpoints (BP1 and BP2) and the distal breakpoint (BP3) in the 15q11-q13 region [51-53]. The typical PWS deletion consists of two classes, Type I and Type II. The larger Type I (TI) deletion involves breakpoint 1 (BP1) which is nearest the centromere while the smaller Type II (TII) deletion involves breakpoint 2 (BP2). Breakpoint 3 (BP3) is located distally in the 15q11-q13 region and is common in both typical deletion subgroups (see Fig. **2**). Butler and colleagues [50] reported that Type I deletions were approximately 6.6 Mb in size and the Type II deletion was 5.3 Mb. There are four recognized genes located between BP1 and BP2 (i.e., *GCP5, CYFIP1, NIPA1, NIPA2*) and individuals were reported recently with behavioral and autistic findings with a deletion involving only the region between BP1 and BP2 [54]. PWS individuals with the larger typical Type I deletion (involving BP1 and BP3) appear to have more behavioral problems such as obsessive compulsion and self-injury along with lower scores on measures of academic performance than seen in those PWS individuals with the smaller Type II deletion (involving BP2 and BP3) [20]. Atypical deletions involving the 15q11-q13 region that are greater or smaller in size than the typical Type I or Type II deletions have been reported in about 5% of PWS individuals [55].

**Fig. (3):**
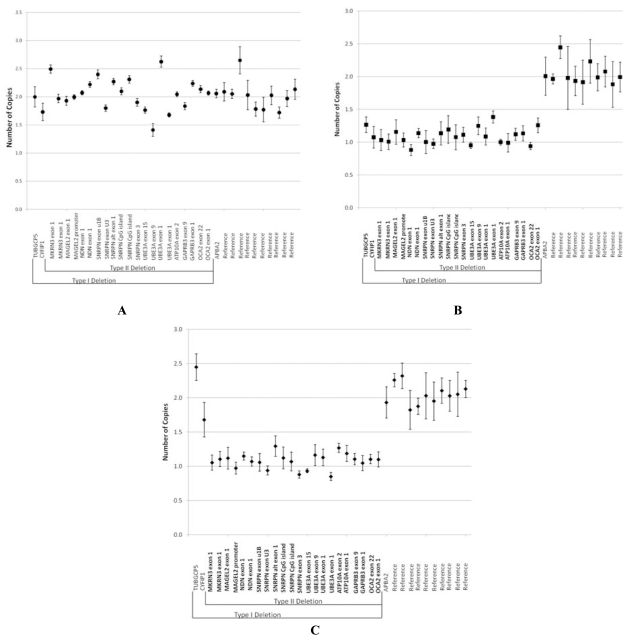
Scatterplot of mean normalized values with standard deviations for probes evaluated by multiplex ligation-dependent probe amplification (MLPA) with methylation-specific analysis. (**A**) Gene copy number of a control individual without any deletion or duplication of probes in the 15q11-q13 region or elsewhere deleted (average copy number of 2.0 for each probe). (**B**) Gene copy number of an individual with Prader-Willi syndrome and the typical Type I deletion of the 15q11-q13 region extending from *GCP5* to *OCA2* with other genes on chromosome 15 (e.g., *APBA2*) or other chromosomes not deleted (copy number of 1.0 = deletion; copy number of 2.0 = non-deletion). (**C**) Gene copy number of an individual with Prader-Willi syndrome and the typical Type II deletion of the 15q11-q13 region extending from *MKRN3* to *OCA2* with other genes on chromosome 15 (e.g., *APBA2*) or other chromosomes not deleted (copy number of 1.0 = deletion; copy number of 2.0 = non-deletion).

**Fig. (4):**
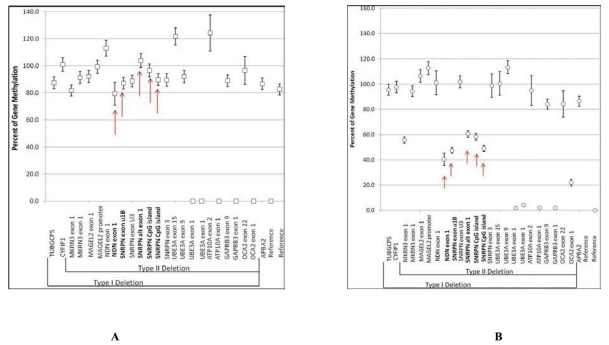
(**A**) Using MLPA and methylation specific probes for imprinted genes from the 15q11-q13 region, the percent of methylation can be measured to determine a normal or abnormal (Prader-Willi syndrome) methylation pattern. If the percentage of methylation for each methylation specific probe (in bold and noted by arrows) falls between 80 to 100 percent, then the diagnosis of Prader-Willi syndrome is made but the methylation pattern will not determine the genetic status (i.e., deletion, maternal disomy or imprinting defect). (**B**) If the percentage of methylation for each methylation specific probe (in bold and noted by arrows) falls between 40 to 60 percent, then the diagnosis is normal (not Prader-Willi syndrome).

There are about 100 genes or transcripts identified in the 15q11-q13 region between BP1 and BP3 and about 10 of these genes are imprinted and paternally expressed [2]. DNA testing to measure the methylation status of genes in the region is quite accurate for diagnosis in about 99% of individuals with PWS. However, this testing will not identify the specific genetic subtype (deletion, maternal disomy or an imprinting defect). Additional testing would be required to determine the genetic subtype, including fluorescence *in situ* hybridization (FISH), genotyping with informative DNA markers, MLPA testing or chromosomal microarray studies [6, 55] (see Figs. **3** and **4**).

The 15q11-q13 region can be divided into four regions; (1) a proximal non-imprinted region between BP1 and BP2 containing the four biparentally expressed genes; (2) a ‘PWS paternal-only expressed region' containing five well characterized protein coding genes (*MKRN3, MAGEL2, NDN*, and the bicistronic *SNURF-SNRPN*), a cluster of five snoRNA genes (*HBII-436, HBII-13, HBII-438, HBII-85* and *HBII-52*) and several antisense transcripts including the antisense transcript to *UBE3A*; (3) an ‘AS region' containing the preferentially maternally expressed genes *UBE3A* and *ATP10A* and (4) a distal non-imprinted region containing a cluster of three GABA receptor genes, the *P* gene for oculocutaneous albinism type 2 (*OCA2*) and the *HERC2* gene [2, 11].

*	SNRPN *(small nuclear ribonucleoprotein N) and a second protein coding sequence (*SNURF*, or *SNRPN* upstream reading frame) as well as multiple copies of C/D box small nucleolar RNAs (snoRNAs) or *SNORDs* involved in RNA

processing and encoded within the *SNURF-SNRPN* transcript are located in the 15q11-q13 region [56]. Other snoRNAs included in this region are *SNORD64, SNORD107, SNORD109A, SNORD115 *and* SNORD116* previously referred to as HBII-13, HBII-436, HBII-438, HBII-52 and HBII-85. The *MKRN3,* *MAGEL2, NDN* and *C15orf2* genes are involved in brain development and function. Exons 4-10 of the complex bicistronic *SNURF–SNRPN* gene encodes for a core spliceosomal protein (SmN) involved in mRNA splicing in the brain, whereas exons 1–3 encode a 71-amino-acid protein enriched in arginine residues [2, 11, 57]. Necdin (*NDN*) is a paternally expressed gene involved with axonal outgrowth and is overly expressed in the hypothalamus, thalamus and pons. Mice deficient for the necdin gene show delayed migration of the sympathetic neurons, neonatal lethality and respiratory problems. The* MAGEL2 *gene is paternally expressed in various brain regions including the hypothalamus and appears to play a role in circadian rhythm, brain structure, behavior, and fertility in humans. The *MKRN3* gene is a member of the makorin (MKRN) RING finger protein gene family which encodes specific proteins (makorins) and abundantly expressed in the central nervous system. Two additional genes (*PWRN1 and PWRN2*) were recently identified and located close to the *NDN* gene. *PWRN1* appears as a novel alternative start site for *SNURF-SNRPN* while *PWRN2* is male germ cell-specific and expressed from the haploid genome [9, 11, 57-59].

The two imprinted genes (*UBE3A, ATP10C*) are maternally expressed or paternally silenced and located in this chromosome region. The *UBE3A* gene causes Angelman syndrome. Other genes located in the distal area of the 15q11-q13 region include several gamma aminobutyric acid (GABA) receptor subunits called *GABRB3, GABRA5, GABRG3* which appear to be paternally biased with loss of the paternal allele resulting in a reduction of expression of greater than 50% [60]. GABA is a major inhibitory neurotransmitter and alterations associated with hunger, obsessive-compulsions and abnormal visual perception and memory; all present in PWS [26]. The *P* gene for pigment production is also located in the distal end of this chromosome region and deleted in both PWS and AS individuals. Mutations of this gene cause albinism.

## GENE/TRANSCRIPT EXPRESSION IN PRADER-WILLI AND ANGELMAN SYNDROMES

To further characterize the genetic findings and identify unique expression patterns in PWS and AS, custom-made cDNA and whole genome-wide expression microarrays were used with RNA isolated from lymphoblastoid cell lines established from three comparison males and seven PWS males with the 15q11-q13 deletion and maternal disomy 15 [61]. More than 47,000 probes were examined and 23,383 were detectable. As expected, imprinted genes from the 15q11-q13 region (e.g, *SNRPN, NDN, MAGEL2*) showed no expression. In addition, there was no difference in expression levels for biallelically expressed genes (e.g., *OCA2*) within the 15q11-q13 region when comparing maternal disomy 15 cell lines with controls. However, two genes previously identified as maternally expressed (*UBE3A,* *ATP10C*) showed a significant increase in expression level in maternal disomy cell lines compared with control or PWS deletion subjects. Several genes/transcripts (e.g., *GABRA5, GABRB3*) showed increased expression in the PWS maternal disomy cells compared with PWS deletion cells, but less than in control cells, indicating a paternal bias of expression [60].

Significant differences (≥ 1.5 fold change with false discovery rate of 20%) were found when placed in functional categories for 323 genes with expression in lymphoblastoid cells from males with PWS relative to comparison males characterized with the Ingenuity Pathway Analysis computer software program. These categories included the nervous system development and function, tissue morphology, hematological system development and function and regulation of gene expression [61]. Genes showing significant changes in expression in the PWS males other than those genes known to be imprinted and paternally expressed included *HCRT* [hypocretin (orexin)], *POMC* (pro-opiomelanocortin, *OXTR* (oxytocin receptor), *MC2R* (melanocortin receptor) and serotonin receptors 3A, 1B and 2B; all are involved with eating behavior in humans. Other genes of interest with reduced expression in PWS individuals included *STAR* (a key regulator of steroid synthesis) and *SAG* (an arrestin family member which desensitizes G-protein-coupled receptors). The impact of the 15q11-q13 genes involved with coding or non-coding protein production on the function of genes located elsewhere in the genome and their role in the causation of PWS will require further investigations.

## ATYPICAL DELETIONS IN PRADER-WILLI SYNDROME: CLINICAL FINDINGS

In 1995, Butler and coworkers [62] reported on a 5-year-old white girl with features of PWS identified during infancy. She was found to have a submicroscopic deletion of the 15q11-q13 region using FISH and DNA marker analysis. The deletion was approximately 100-200 kb in size and showed a *de novo* paternal deletion of the imprinting center and the *SNRPN* gene region including *SNORD116*. She developed a milder PWS phenotype with decreased growth, cognitive and behavioral problems than typically seen in PWS. She also had normal pigmentation.

More recently, a female patient with an atypical deletion of the chromosome 15q11-q13 region was reported due to an unbalanced chromosome 15 translocation involving only a paternal deletion of the *MKRN3, MAGEL2* and *NDN* genes [63]. She presented with obesity, mental retardation and a high pain threshold but lacked other features of PWS. Other patients included a male child with several features of PWS reported by Sahoo and colleagues in 2008 [64]. The features included neonatal hypotonia, feeding problems, obesity, and hypogenitalism, but with increased birth weight, a large head and facial appearance not consistent with PWS. Genetic testing showed a paternal deletion of snoRNA *SNORD116* and part of the *SNORD115* gene cluster. Smith and colleagues [65] reported a 19-year-old male with hyperphagia and severe obesity, mild intellectual disability and hypogonadism with a 187 kb deletion which included *SNORD116*. Deletions involving the proximal long arm of chromosome 15 can be smaller or larger than the typical deletion in individuals with features of PWS as supported by a recent report by Butler and colleagues [66] of an infant girl with a *de novo* interstitial 15q11-q14 deletion, which is larger than the typical deletion. She presented with hypotonia, a poor suck, feeding problems, mild micrognathia, preauricular ear tags, a high arched palate, edematous feet and coarctation of the aorta; several of these features are not typical for PWS but reported previously in individuals with this expanded deletion of chromosome 15.

Clinical reports of atypical deletions (larger and smaller) give further insight into the genes causing PWS. The very small atypical deletions appear to contribute to several PWS features, specifically the paternally expressed snoRNAs including *SNORD115* and *SNORD116*. In addition, mice with the snoRNA equivalent of *SNORD116* removed causes hyperphagia and growth deficiency. Furthermore, Kishore and Stamm [67] reported that the paternally expressed snoRNA *SNORD115* regulates alternative splicing of the serotonin 5-HT2C receptor leading to an abnormal receptor involved with eating behavior. Therefore, PWS appears not to be caused by a single locus or gene defect, but by a deficiency of a combination of imprinted genes in the 15q11-q13 region including *SNURF-SNRPN* and the *SNORD* genes [64]. Deficiencies of other imprinted genes in the region, including *MKRN3, MAGEL2* and/or *NDN* apparently play a role but not sufficient alone to generate the cardinal features recognized in Prader-Willi syndrome.

## CLINICAL COMPARISON OF GENETIC SUBTYPES IN PRADER-WILLI SYNDROME

Individuals with PWS who have the typical chromosome 15 deletion are hypopigmented and more homogeneous in their clinical presentation [1, 13]. Those with maternal disomy have fewer typical facial features and are less likely to have certain behavioral findings such a skin picking, unusual skill with jigsaw puzzles, a high pain threshold and articulation problems. PWS individuals with maternal disomy are usually diagnosed later, which may reflect a milder phenotype. PWS individuals with the 15q11-q13 deletion have a greater number of compulsive symptoms than those with maternal disomy 15 [47, 68]. Furthermore, PWS children with the deletion possess relative strengths in standardized visual-spatial tasks such as object assembly [28].

Measures of intellectual function and academic achievement in PWS have shown that those with maternal disomy have significantly higher verbal IQ scores than those with the typical deletion [27]. However, PWS deletion individuals score higher on object-assembly subtests, supporting specific visual perceptual skills as relative strengths in these individuals. Discrimination of shape and motion testing and kinetic form visual performance in the maternal disomy group are generally worse than seen in the deletion individuals. However, the superior visual recognition memory is greater in the maternal disomy individuals [26].

To further characterize clinical differences in the genetic subtypes in PWS, Butler and colleagues [20] reported differences in psychological, cognitive and behavioral data collected from young adults with PWS representing the longer Type I deletion involving breakpoints BP1 and BP3 versus those with the shorter Type II deletion involving BP2 and BP3. PWS individuals with Type I deletions scored significantly worse in self-injurious and maladaptive behavior assessments compared with Type II deletions. Obsessive–compulsive behavior was also more common in Type I deletions. Adaptive behavior scores were generally more abnormal including lower reading and math skills and more compulsions in Type I versus Type II deletion individuals. Intellectual ability and academic achievement were lower in individuals with Type I deletion (see Table **1**).

To help explain the genetic deletion subtype differences, Bittel and colleagues [69] studied expression patterns of four genes (*GCP5, CYFIP1, NIPA2, NIPA1*) located between BP1 and BP2 and correlated the expression with behavioral, maladaptive and intellectual ability scores in the two PWS deletion groups. Three of these genes are implicated in central nervous system development. Correlation studies with gene expression and behavioral and cognitive scores indicated a direct relationship between the quantity of gene message and higher assessment scores. The coefficient of determination indicated the quantity of messenger RNA of the four genes explained 24% to 99% of the variation of the behavioral and academic assessment scores, with *NIPA2* having the greatest impact.

In summary, strides are being made to better understand the role of genetics and impact on clinical care in PWS and other genetic conditions. The publication of Health Supervision for Children with Prader-Willi syndrome published in Pediatrics [70] and the use of interactive computer websites such as the Prader-Willi Syndrome Diagnosis Module in the Medical Home Portal (www. med.calhomeportal.org) can provide useful information to care providers treating individuals with PWS. Other internet sites include the Prader-Willi Syndrome Association (USA) at www. pwsausa.org; Foundation for Prader-Willi Research at www.fpwr.org; Foundation for Prader-Willi Research Canada at www.onesmarcstep.ca and the International Prader-Willi Syndrome Organization at www.ipwso.org. These are excellent online sources of information for the parents and health care providers needed for management and care of individuals at all ages with Prader-Willi syndrome.

## Figures and Tables

**Table 1:** Reported Clinical Differences in Genetic Subtypes of Prader-Willi Syndrome

Genetic defect	Characteristics
Typical 15q11-q13 deletion	Hypopigmentation and more homogenous clinical findings including a typical facial appearance; more self-injurious behavior (skin picking); higher pain threshold; greater jigsaw puzzle skills than seen in maternal disomy
*Type I deletion*	Increased maladaptive and compulsive behavior relative to Type II deletion and maternal disomy; poorer academic performance relative to Type II deletion and maternal disomy
*Type II deletion*	Better adaptive behavior and social skills relative to Type I or maternal disomy
Maternal disomy 15	Higher verbal IQ scores; greater numeric calculation skills, superior visual memory, poorer object assembly and visual perceptual skills; increased psychosis relative to typical deletion

## ABBREVIATIONS

PWS: = Prader-Willi syndrome
AS: = Angelman syndrome
DNA: = Deoxyribonucleic acid
IQ: = Intelligence quotient
UPD: = Uniparental disomy
fMRI: = Functional magnetic resonance imaging
MLPA: = Multiplex ligation-dependent probe amplification
